# Preventive Cognitive Therapy versus Treatment as Usual in preventing recurrence of depression: protocol of a multi-centered randomized controlled trial

**DOI:** 10.1186/s12888-015-0508-8

**Published:** 2015-07-01

**Authors:** Margo de Jonge, Claudi LH Bockting, Martijn J Kikkert, Judith E Bosmans, Jack JM Dekker

**Affiliations:** Department of research Arkin, Klaprozenweg 111, 1033 NN Amsterdam, The Netherlands; Department of Clinical Psychology, Utrecht University, Heidelberglaan 1, 3584 CS Utrecht, The Netherlands; Department of Health Sciences and the EMGO Institute for Health and Care Research, Faculty of Earth and Life Sciences, Vrije Universiteit, De Boelelaan 1085, 1081 HV Amsterdam, The Netherlands

**Keywords:** Depression, Relapse, Recurrence, Cognitive Therapy, Prevention

## Abstract

**Background:**

Major depressive disorder (MDD) is projected to rank second on a list of 15 major diseases in terms of burden in 2030. The contribution of MDD to disability and health care costs is largely due to its highly recurrent nature. Therefore, part of the efforts to reduce the disabling effects of depression should focus on preventing recurrence, especially in patients at high risk of recurrence. The best established effective psychological intervention is cognitive therapy, with indications for prophylactic effects after remission.

**Methods/Design:**

In this randomized controlled trial (cost-) effectiveness of Preventive Cognitive Therapy (PCT) after response to Acute Cognitive Therapy (A-CT) will be evaluated in comparison with Treatment As Usual (TAU). Remitted patients that responded to A-CT treatment with at least two previous depressive episodes will be recruited. Randomization will be stratified for number of previous episodes. Follow-ups are at 3, 6, 12 and 15 months. The primary outcome measure will be the time to relapse or recurrence of depression meeting DSM-IV criteria for a major depressive episode on the Structured Clinical Interview for DSM-VI Axis I Disorders (SCID-I). Costs will be measured from a societal perspective.

**Discussion:**

This study is the first to examine the addition of PCT to TAU, compared to TAU alone in patients that recovered from depressive disorder with A-CT. Alongside this effect study a cost effectiveness analysis will be conducted. Furthermore, the study explores potential moderators to examine what works for whom.

**Trial registration:**

Netherlands Trial Register (NTR): 2599, date of registration: 11-11-2010.

## Background

High prevalence, and frequent relapse and recurrence contribute to the public health significance of Major Depressive Disorders (MDD). Depression is the most disabling disorder worldwide measured in years lived with disability [1]. In various recent epidemiological studies, the annual prevalence in the general population varies from 4 % to 6 % [2, 3]. Epidemiological estimates place the lifetime prevalence of MDD at more than 16 % [4]. However, the major contribution of MDD to disability and health care costs is largely due to its highly recurrent nature. The large majority of individuals with MDD experience more than one episode and the probability of another episode increases with each relapse or recurrence [5, 6]. Reported relapse and recurrence rates for high risk groups, rise up to 60–70 % recurrence over 2 year [7, 8]. Apart from the number of previous episodes, residual symptoms after remission are risk factors for recurrence [9, 10]. Optimizing long-term outcomes is therefore an important goal in the treatment of MDD, especially for well-known high risk groups, i.e. patients with multiple previous episodes and/or residual symptoms.

We know that acute phase treatment of depression with Cognitive Therapy (A-CT) has a prophylactic effect [11]. After completing A-CT, patients are less likely to relapse than after stopping a similar course of pharmacotherapy [12]. However, recent meta-analysis showed that after remission on A-CT, many responders still have a relapse-recurrence (29 % within 1 year and 54 % within 2 years) [8]. Other studies also show that after remission a sizable number (40 %) will experience a return of symptoms after treatment [9, 13].

The most commonly used treatment to prevent relapses and recurrences is antidepressant medication (AD). However, 70–80 % of patients are not willing to take AD for a long period of time [14, 15]. Also non-adherence is common among AD-users, which is troubling as patients’ protection from recurrence ceases on discontinuation of AD and therefore increases the risk of recurrence [16, 17]. Most patients prefer psychological interventions to drug treatment, this is especially relevant in long term treatment as studies show that patients’ treatment preference is associated to compliance [18].

Jarrett and colleagues developed and tested a model of Continuation-phase Cognitive Therapy (C-CT). C-CT consists of ten sessions over a period of eight months and is provided after 20 sessions of A-CT, both provided by the same therapist. They found that C-CT reduces time to relapse and recurrence compared with an assessment-only control [19]. Responders to A-CT who received C-CT were significantly less likely to relapse (10 %) compared to those who received the assessment-only control (31 %). In the same study responders to A-CT with unstable remissions also benefited (37 % vs 62 %). Furthermore, responders to A-CT with more residual symptoms were more likely to require C-CT to avoid relapse than responders with lower or no residual symptoms [20]. Although these results are promising they are not replicated yet. Given the limited therapy resources, and the fact that remitted patients might not be motivated to an intervention that consists of eight months after remission, C-CT with fewer sessions given within a two month time-period would be desirable.

Building on this knowledge Bockting developed Preventive Cognitive Therapy (PCT). PCT is a form of Cognitive Therapy aimed at treating an underlying vulnerability and thereby inducing long-term effects on depression recurrence. After remission, sequential PCT is proven effective in preventing relapse and recurrences in patients with multiple episodes over 5.5–10 years compared to treatment as usual (TAU) [7, 21, 22]. However, this study did not include patients treated with A-CT. Therefore, the (cost-) effectiveness of PCT after remission on A-CT is still unknown and the main goal of this study. PCT differs from C-CT as it is a brief intervention, only taking 8 weeks, and can be given by another therapist than the one providing A-CT. Because PCT is shorter and given within two months this might enhance possibilities for dissemination.

This study is the first to assess the (cost-) effectiveness of 8 sessions of PCT in comparison with TAU in high risk patients who remitted after receiving A-CT. A clinically relevant difference between PCT and TAU might result in enormous health effects on the population level.

### Trial objectives

In this study, the addition of PCT to TAU will be compared to TAU alone in patients that recovered from recurrent depressive disorder after treatment with A-CT. Alongside this effectiveness study a cost-effectiveness study will be done. It is hypothesized that adding PCT to TAU is clinically superior tot TAU alone in preventing relapse and recurrence of depressive disorder.

Furthermore, the study explores potential moderators to examine what works for whom. Potential mediators will also be examined to explore the working mechanism of PCT by assessing coping, social support, dysfunctional attitudes, affiliation, daily stress and functioning, and their association to risk of relapse in depression, before, during and after treatment.

## Methods/Design

### Design

In this multi-center randomized controlled trial with a follow-up of 15 months, we compare the addition of PCT to TAU in patients who recovered from depressive disorder after receiving CT, to TAU alone. An economic evaluation from a societal perspective will be performed alongside this RCT.

The target population consists of patients with a history of two or more depressions which is a group at elevated risk of relapse and recurrence of depression. This group consumes a considerable amount of healthcare mainly due to relapse rates witch rise which increasing numbers of previous episodes as identified in several studies [10].

**Fig. 1:**
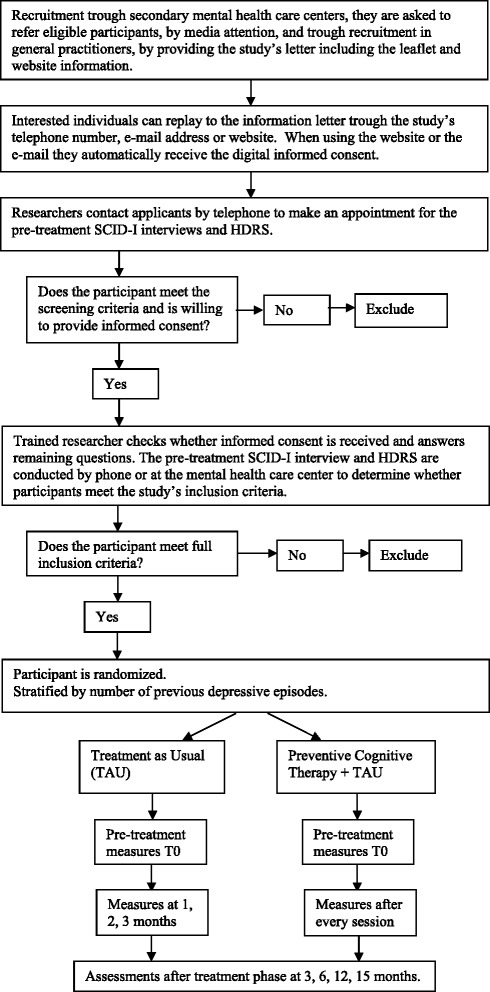
Flow-chart of the study

Randomization at patient level will be undertaken by an independent researcher using a computer-generated random schedule and will be stratified by number of previous depressive episodes. The randomization is accessible solely by the independent researcher. For a Flow diagram of the assessment methods see Fig. 1.

### The interventions

#### Acute Cognitive Therapy

All patients who enter this study have received Acute Cognitive Therapy (A-CT) during the acute phase of depression consisting of at least 8 sessions specifically designed for patients with depression. A-CT is directed at the identification of maladaptive cognitions. Each session follows a fixed structure, with agenda setting, review of homework, explanation of rationale of each session, and assignment of homework [23].

#### Treatment as usual

TAU consists of usual care that patients receive in primary care and in secondary care, after A-CT for depression. TAU may include no treatment at all or anti-depressant maintenance medication, which is in most cases provided by the general practitioner (GP). Thus, use of AD will not be an exclusion criterion. Treatment received as part of TAU will be included as covariate in the final analysis. A comparison of the intervention with TAU is relevant from a public health perspective since it would help to demonstrate the intervention’s added value over and above TAU. We will not intervene with TAU, but closely monitor healthcare utilization in TAU.

#### Preventive Cognitive Therapy

After response to A-CT, there will be a minimum two month waiting period before PCT will start. PCT consists of eight individual sessions once a week, offered as sequential treatment after response to A-CT. PCT is an adapted type of CT specifically developed to prevent relapse in recurrent depression. Similar to regular CT, each PCT session follows a fixed structure, with agenda setting, review of homework, explanation of rationale of each session, and assignment of homework [24].

PCT differs from A-CT in several aspects. Unlike A-CT, PCT is not primarily directed toward modifying negative thoughts. Instead, it starts with the identification of negative thoughts and dysfunctional attitudes and beliefs, aided by a self-report questionnaire with examples of attitudes and specific techniques such as the downward arrow technique. The focus of treatment is then directed on examining these attitudes using different cognitive techniques such as Socratic questioning and identification of positive phantasy attitudes. Moreover, patients are encouraged to practice with alternative attitudes in the final sessions. In addition, unlike with traditional A-CT, specific attention will be paid to enhancing the memory and retrieval of positive experiences and making a personal prevention plan [21]. A specific manual for the client and therapist has been published describing the structure of the treatment [24].

### Therapists

Therapists in the PCT condition are trained psychologists who are specialized in CBT. They will receive a one day training in PCT from the author of the PCT protocol that is used in this study [24]. During the study the therapists participate in regular supervision groups led by the trainer, as well as peer supervision groups led by the principal investigator. Regular supervision will be held once a week, the peer supervision once every four months. The therapists who perform the PCT are not the same therapists that provided the A-CT.

### Sample size

In total, 214 patients will be recruited. This sample size is needed to detect a difference in the primary outcome (cumulative proportion of relapse/recurrence rates over 15 months) of at least 20 % between the conditions in a 1-sided test at alpha = 0.05 and a power of (1-beta) = 0.80. For this we need 85 participants in each condition. Allowing for a drop out of 20 % over the two years, we need to include 107 participants in each condition at baseline.

### Referral and recruitment

The first recruitment strategy is via secondary mental healthcare facilities. Patients who are currently treated for depression with cognitive therapy will be informed by their therapist about this study. Patients will also be offered written information by either a research associate or via e-mail or post. Secondly, patients who have been treated for depression with A-CT in the past will be informed through their former mental healthcare facility. They will receive written information about the study and a request to sign the informed consent. The third recruitment strategy is via media. The study will be promoted through press releases to local media, advertisements and digital online promotions using the study’s website. If someone is interested in participating, s/he can contact the researcher by e-mail, phone or through the website.

### Informed consent

We inform patients about the study before they sign informed consent. They will receive a letter containing all the information and a digital informed consent. If they agree to enter the trial, they fill out the digital informed consent and then are contacted by a researcher for the baseline assessment.

Approval of the Stichting Medisch-Ethische Toetsingscommissie Instellingen Geestelijke Gezondheidszorg (METiGG) is obtained and the trial is conducted in compliance with the Declaration of Helsinki [25].

### Inclusion criteria

We will include patients:

- with at least two previous depressive episodes
- who are currently in remission according to DSM-IV criteria, for at least two months as assessed by the Structured Clinical Interview for DSM-IV Axis I Disorders (SCID-I)
- with none to mild depressive symptoms defined as a current score of <14 in the 17 item Hamilton Depression rating scale
- who have received A-CT, with a minimum of eight sessions
- who are fluent in Dutch

### Exclusion criteria

We exclude patients who have:

- current mania or hypomania or a history of bipolar illness, any psychotic disorder (current and previous)
- current alcohol or drugs misuse
- acute predominant anxiety disorder

There are no restrictions with respect to co-morbidity on Axis II and III, in exception of a concurrent chronic somatic illness that is as risk factor for relapse and recurrence.

### Withdrawal

Participants can withdraw from treatment or from the study at any time. If they decide to withdraw from the trial treatment we ask them whether they are willing to finish the remaining assessments.

### Assessment of eligibility and baseline measures

After filling in the informed consent participants are contacted by a researcher for baseline assessment. This assessment is done at the secondary care facility, by phone or at the participant’s home, depending on their preference. The researcher will check for potential inclusion and exclusion criteria through an initial screening. They will start the baseline assessment using the Structured Clinical Interview for DSM-IV Axis I Disorders (SCID-I) [26] and Hamilton Depression rating scale (HAM-D) [27]. Afterwards the researcher contacts the primary investigator to evaluate the participant and decide whether the participant can participate in the study. If the participant cannot participate in the study, s/he will be contacted by the researcher. If the participant is eligible to participate in the study, s/he will be contacted by the primary investigator to receive the allocation. They will also receive the self-report measures completing the baseline measure by e-mail. After 3 months, all participants received a one year magazine subscription. For a complete overview of the procedure see Fig. 1.

### Outcome measures/Instruments

Every participant will be monitored over a period of 15 months. After baseline assessment, follow up assessments of patients in the TAU condition will be performed at 1, 2, 3, 6, 12 and 15 months. Patients in the experimental condition will be assessed every session and after 3, 6, 12, and 15 months. The assessments done at baseline and at 15 months consist of an interview and self-report measures. Al interviews will be performed by trained assessors. The assessors are blind to the allocation of the participants. The remaining assessments are self-report questionnaires, which are filled in by the patients and their therapists. For an overview of the assessments at baseline, post treatment and follow up see Table 1. For an overview of the assessments after each session see Table 2.

**Table 1:** Overview of assessments

Measure	Description	T0	T1	T2	T3	T4	T5	T6^a^
Interviews								
SCID-I	DSM-IV-TR Axis I disorders	x						x
HAM-D	Depressive symptoms and severity	x						x
Self report measures								
IDS-SR	Depressive symptoms	x			x	x	x	x
IIP-C	Social and interpersonal functioning	x						
EQ-5D	Quality of life	x			x	x	x	x
TIC P	Direct/indirect costs	x			x	x	x	x
PMS	Psychological mindedness	x				x		
PDQ-4	Personality				x			
Brugha	Life events	x						x
EPCL	Everyday problems	x				x	x	
Mastery scale	Mastery	x			x	x	x	x
VAMS	Mood	x			x	x	x	x
IVM	Victimization							x
SSL-I	Social support		x	x	x	x		x
UCL	Coping		x	x	x	x		x
AAQ	Experiential acceptance and avoidance		x	x	x			
ORS	Functioning		x	x	x			
Affiliation	Affiliation		x	x	x			
PANAS-X	Emotions		x	x	x			
DAS	Dysfunctional attitudes		x	x	x		x	
BDI	Depressive symptoms		x	x	x			

^a^T0 = Baseline, T1 = 1 month, T2 = 2 months, T3 = 3 months, T4 = 6 months, T5 = 12 months, T6 = 15 months

**Table 2:** Overview of assessments after each session

Measure	Description	S1	S2	S3	S4	S5	S6	S7	S8^a^
UCL	Coping	x		x		x		x	
SSL-I	Social support		x				x		
AAQ	Experiential acceptance and avoidance		x				x		
ORS	Functioning	x	x	x	x	x	x	x	x
SRS	Session evaluation		x		x		x		x
BDI	Depressive symptoms	x		x	x	x		x	x
WAI-SR	Therapeutic relationship		x		x		x		x
DAS-17	Dysfunctional attitudes	x	x	x	x	x	x	x	x
Affiliation	Affiliation		x		x				x
Daily h.	Daily stressors	x		x		x		x	
PANAS	Emotions	x	x	x	x	x	x	x	x

^a^S = session number

### Primary outcome

The primary outcome measure will be the cumulative proportion of relapse or recurrence of depression meeting DSM-IV criteria for a major depressive episode according to the Structured Clinical Interview for DSM-VI Axis I Disorders (SCID-I) over a follow-up period of 15 months.

### Secondary outcome

Secondary outcome variables are;

- Depressive symptom severity as measured with the HAM-D [27], Inventory of Depression Symptomatology (IDS) [28] and the Beck Depression Inventory (BDI-II) [29].
- Duration of relapse/recurrence.
- Number of relapses.
- Quality of life as measured by the EuroQol (EQ-5D). The EQ-5D scores will be used to calculate utilities using the Dutch tariff [30]. Quality-adjusted life-years (QALYs) will be calculated by multiplying the utility of a health state by the time spent in this health state using linear interpolation between time points. Higher QALY scores indicate higher quality of life.
- Health care utilization and productivity losses will be measured with an adapted version of the Trimbos Questionnaire for Costs associated with Psychiatric illness (TIC-P), a widely used health service receipt interview in economic evaluations [31]. Costs that will be included in the economic evaluation are direct medical and non-medical costs, and indirect costs. Resource utilization (GP visits, hospital days, ect.) will be valued using Dutch standard costs [32]. Using the TiC-P, both absenteeism and presenteeism (working less efficiently while at work) will be measured. Productivity losses will be valued using the friction cost method.
- The frequency and intensity of daily stress as measured with the Brugha and the Everyday Problem Checklist (EPCL) [33].

### Other parameters

As potential moderators, mediators and mechanisms of change we will analyze; personality traits measured by the Personality Diagnostic Questionnaire (PDQ-4) [34], psychological mindedness measured by the Psychological Mindedness Scale (PMS) [35], mastery measured by the Mastery scale [36], social support measured by the Sociale Steun Lijst (SSL-I) [37], coping measured by the Utrechtse Coping Lijst (UCL) [38], experiential acceptance and avoidance measured by the Acceptance and Action Questionnaire (AAQ-II) [39], functioning measured by the Outcome Rating Scale (ORS) [40], affiliation measured by the Affiliation scale [41], emotions measured by the Positive and Negative Affect Schedule (PANAS-X) [42], dysfunctional attitudes measured by the Dysfunctional Attitude Scale (DAS) [43, 44], social en interpersonal functioning measured by the Inventory of Personal Problems (IIP-C) [45], depressive mood measured by a one item mood scale [46], session evaluation measured by the Session Rating Scale (SRS) [47], therapeutic relationship measured by the Working Alliance Inventory-Short Revised (WAI-SR) [48], mood measured by a one-item Visual Analogue Mood Scale (VAMS) [46] and Victimisation measured by the Dutch version of the Integral Safety/Security Monitor (IVM) [49].

### Analysis

The primary outcome measure will be the time to relapse or recurrence meeting DSM-IV criteria for a major depressive episode on the Structured Clinical Interview for DSM-VI (SCID-I). Occurrence of relapse or recurrence (current or since the last assessment point) will be assessed after 15 months, by trained psychologists who are blind to the treatment condition. Cox regression analysis will be performed. Analysis will be conducted by intention to treat, including all subjects randomized in the study, and per protocol. Statistical significance will be set at p < .05. The time (in weeks) of relapse or recurrence to Major Depression, as defined above, will be the dependent variable in survival analysis. The treatment group will be used as predictor, and the stratification variable will be used as covariate. In addition, time since previous episode and time since last treatment will be included as covariates.

In addition, we use the HAM-D, to assess the severity of depression at both time points, together with the SCID-I.

Mixed-model analysis will be used for the quantitative measures. As covariates we will use the stratification variable (number of episodes). Potential moderators to be examined include gender, residual symptoms of depression, duration of remission, duration of last episode, coping, age of onset and current use of AD.

For the economic evaluation the relationship between costs and health outcomes of the two treatment conditions will be evaluated using a societal perspective. Both a cost-effectiveness analysis with depression-free survival time as effect measure and a cost-utility analysis with QALYs as effect measure will be performed.

The economic evaluation will be done according to the intention-to-treat principle. Incremental cost-effectiveness ratios (ICERs) will be calculated by dividing the difference in mean total costs between the treatment groups by the difference in mean effects between the treatment groups. Bootstrapping with 5000 replications will be used to estimate 95 % confidence intervals around cost differences and the uncertainty surrounding the ICERs. Rubin’s rules will be used to pool the results from the different multiply imputed datasets. Uncertainty surrounding the ICERs will be graphically presented on cost-effectiveness planes. Cost-effectiveness acceptability curves showing the probability that the intervention is cost-effective in comparison with usual care for a range of different ceiling ratios will also be estimated [50].

## Discussion

High prevalence and frequent relapse and recurrence contribute to the public health significance of MDD. MDD is considered the most disabling disorder worldwide measured in years lived with disability [1]. This trial will be the first to compare short term individual PCT to TAU following A-CT in patients with major depression. Apart from the evaluation of the effectiveness and cost-effectiveness, we examine what works for whom, by measuring potential mediators and moderators after each PCT session and throughout the entire study.

Recent studies show that although long-term continuation of AD is likely to be effective in preventing relapse and recurrence, patients prefer psychological therapies above taking ADs [14, 51]. Therefore, sequential PCT following remission on A-CT may be a good alternative or addition to TAU. Considering the substantial negative effects on quality of life and the increased societal costs associated with depression there is a need for short-term and cost-effective interventions that can be provided by any psychologist. The results of this study might improve better treatment matching by examining mediators and moderators and thereby clarifying what is best for whom. In addition, mediation variables will be examined to get more insight into the essential ingredients of the preventive CT used. This trial aims to contribute to the improvement of more efficient therapeutic interventions to prevent relapse and recurrence in depression.
